# The Musashi proteins MSI1 and MSI2 are required for photoreceptor morphogenesis and vision in mice

**DOI:** 10.1074/jbc.RA120.015714

**Published:** 2020-11-22

**Authors:** Jesse Sundar, Fatimah Matalkah, Bohye Jeong, Peter Stoilov, Visvanathan Ramamurthy

**Affiliations:** 1 Department of Biochemistry, Robert C. Byrd Health Sciences Center, West Virginia University, Morgantown, West Virginia, USA; 2 Department of Ophthalmology and Visual Sciences, Robert C. Byrd Health Sciences Center, West Virginia University, Morgantown, West Virginia, USA; 3 Department of Neuroscience, Robert C. Byrd Health Sciences Center, West Virginia University, Morgantown, West Virginia, USA

**Keywords:** Msi1, Msi2, Musashi, retina, photoreceptor, RNA-binding protein, splicing, CC, connecting cilium, ERG, electroretinographic, INL, inner nuclear layer, MAK, male germ cell–associated kinase, MSI1, Musashi-1, MSI2, Musashi-2, ONL, outer nuclear layer, PBST, PBS supplemented with 0.1% Triton X-100, PDE6β, phosphodiesterase-6β, P, postnatal day, PNA, peanut agglutinin, PRPH2, peripherin-2, RBDs, RNA-binding domains, TTC8, tetratricopeptide repeat domain 8

## Abstract

The Musashi family of RNA-binding proteins is known for its role in stem-cell renewal and is a negative regulator of cell differentiation. Interestingly, in the retina, the Musashi proteins MSI1 and MSI2 are differentially expressed throughout the cycle of retinal development, with MSI2 protein displaying robust expression in the adult retinal tissue. In this study, we investigated the importance of Musashi proteins in the development and function of photoreceptor neurons in the retina. We generated a pan-retinal and rod photoreceptor neuron-specific conditional KO mouse lacking MSI1 and MSI2. Independent of the sex, photoreceptor neurons with simultaneous deletion of *Msi1* and *Msi2* were unable to respond to light and displayed severely disrupted photoreceptor outer segment morphology and ciliary defects. Mice lacking MSI1 and MSI2 in the retina exhibited neuronal degeneration, with complete loss of photoreceptors within 6 months. In concordance with our earlier studies that proposed a role for Musashi proteins in regulating alternative splicing, the loss of MSI1 and MSI2 prevented the use of photoreceptor-specific exons in transcripts critical for outer segment morphogenesis, ciliogenesis, and synaptic transmission. Overall, we demonstrate a critical role for Musashi proteins in the morphogenesis of terminally differentiated photoreceptor neurons. This role is in stark contrast with the canonical function of these two proteins in the maintenance and renewal of stem cells.

In eukaryotes, alternative splicing of pre-mRNA increases protein diversity and controls gene expression. Diversification of proteomes through alternative splicing is a defining characteristic of metazoans and was expanded dramatically in bilaterians (1). Alternative splicing is prevalent in vertebrate neurons and is critical for the development and function of vertebrate nervous systems (2, 3, 4, 5, 6, 7).

We previously showed that photoreceptor neurons exploit a unique splicing program (8). Motif enrichment analysis suggested that Musashi-1 (MSI1) and Musashi-2 (MSI2) promote the use of photoreceptor-specific exons (8). We further showed that MSI1 is critical for utilization of photoreceptor-specific exon in the *Ttc8* gene (8). In addition, Musashi promotes the splicing of several photoreceptor-specific exons when overexpressed in cultured cells (8). Recently, analysis of a comprehensive gene expression data set demonstrated that photoreceptors utilize a unique set of alternative exons that are primarily regulated by MSI1 and MSI2 (9).

The MSI1 and MSI2 proteins have two highly conserved RNA-binding domains (RBDs) in the N-terminal region, which show close to 90% sequence identity and recognize a similar UAG motif in RNA (10). The two RBDs of MSI1 and MSI2 are followed by a less-conserved C-terminal region that shows approximately 70% sequence identity (11). The high degree of sequence identity between the MSI1 and MSI2 results in functional redundancy between the two proteins (12, 13).

Vertebrate photoreceptors are neurons specialized in detecting and transducing light stimuli. Photoreceptors are characterized by segmented morphology that compartmentalizes phototransduction, core cellular functions, and synaptic transmission. The light sensing machinery is confined to the outer segment (OS), a stack of membranes that is elaborated by cell’s modified primary cilium. The OS is a dynamic structure that is remade every 7 to 10 days. Consequently, maintenance of the OS requires a high rate of transport of membranes and proteins through the connecting cilium (CC) (14).

The predicted splicing targets of Musashi in photoreceptors include pre-mRNAs from ciliary (*Ttc8, Cep290, Cc2d2a, Prom1*) and synaptic transmission–associated genes (*Cacna2d4, Slc17a7*) (15, 16, 17, 18, 19, 20, 21). These genes are crucial for photoreceptor development and function (15, 16, 17, 18, 19, 20, 21). We proposed that production of photoreceptor-specific splicing isoforms that is promoted by Musashi is necessary for the development and maintenance of photoreceptor cells *in vivo* (8).

To test if Musashi drives photoreceptor development and function, we removed *Msi1* and *Msi2* in the developing retina and rod photoreceptor cells. We find that Musashi proteins are essential for photoreceptor function, morphogenesis, and survival but not their specification. Specifically, the Musashi proteins are crucial for the OS and axoneme development. As expected, disruption of the Musashi genes led to the loss of expression of photoreceptor-specific splicing isoforms.

## Results

### Validation of the conditional KO mouse models

**Figure 1:**
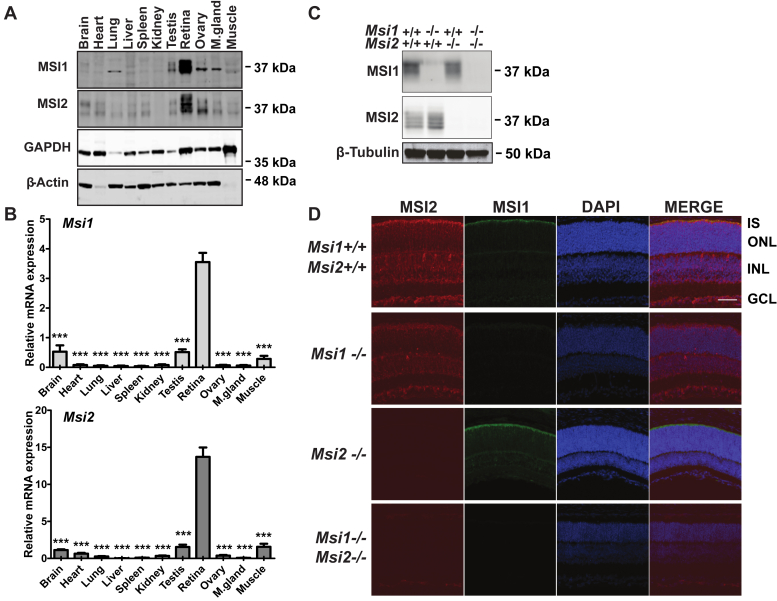
**Conditional Musashi KO mouse models.***A*, immunoblot of indicated tissues from adult WT mice probed with MSI1 and MSI2 antibodies. GAPDH and β-Actin serve as a loading control. *B*, relative Msi1 and Msi2 transcript levels determined by quantitative RT-PCR. Expression in the retina is significantly higher that that in other tissues: one-way ANOVA with Welch’s correction F(10, 8.73) = 9.66, *p* = 0.001 for Msi1 and F(10, 8.59) = 21.02, *p* < 0.001 for Msi2. Significance levels of pairwise *t*-test after FDR correction are indicated as follows: ∗∗∗ = *p*-value < 0.001. *C*, western blot analyses of Musashi in retinal lysates from ret-Msi1-/-, ret-Msi2-/-, and ret-Msi1-/-: Msi2-/- mice at P10. β-tubulin levels provide a loading control. *D*, retinal sections from ret-Msi1-/-, ret-Msi2-/-, and ret-Msi1-/-: Msi2-/- mice at P10 probed with MSI1 (*green*) and MSI2 (*red*) antibodies along with a DAPI nuclear counterstain (*blue*). Scale bar = 50 μm. DAPI, 4′,6-diamidino-2-phenylindole; FDR, false discovery rate; GCL, ganglion cell layer; INL, inner nuclear layer; IS, inner segment; ONL, outer nuclear layer; P, postnatal day.

We analyzed the expression of Musashi proteins in various tissues from adult mice. Of all the tissues we tested, the retina showed the highest expression of MSI1 and MSI2 proteins (Fig. 1*A*), in line with the previously reported high transcript levels for *Msi1* and *Msi2* in rod photoreceptors (9). Quantitative RT-PCR of *Msi1* and *Msi2* levels in the tissues shown in Figure 1*A* further confirmed significantly higher levels of expression of the two transcripts in the retina (Fig. 1*B*). To test the biological significance of Musashi protein expression in the murine retina, we used *Cre-LoxP* conditional recombination to remove *Msi1*, *Msi2*, or both the *Msi1* and *Msi2* genes throughout the entire retina and ventral forebrain using the *Six3-Cre* transgene (22). Throughout this work, we refer to *Musashi* floxed mice that are hemizygous for the *Six3-Cre* transgene as *ret-Msi-/-* mice. The conditional recombination results in the deletion of *Msi1’s* transcription start site, exon 1, and exon 2 (13). For *Msi2*, the transcription start site and the first four exons are removed after Cre-mediated recombination (13). The ablation of MSI1 and MSI2 was confirmed by immunoblotting retinal lysates from KO mice at postnatal day 10 (P10) (Fig. 1*C*). Immunofluorescence microscopy of retinal cross sections obtained from the KO mice affirmed the absence of MSI1 and MSI2 expression in the retina (Fig. 1*D*).

### The Musashi proteins are crucial for photoreceptor function

**Figure 2:**
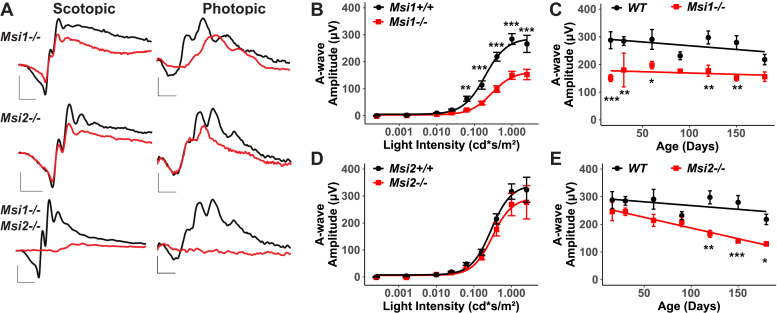
**The Musashi proteins are crucial for normal visual response.***A*, representative scotopic and photopic electroretinograms (ERGs) from the ret-Msi1-/-, ret-Msi2-/-, and ret-Msi1-/-: Msi2-/- mice at P16. Scotopic ERGs were obtained after overnight dark adaptation using 0.151 cd-s/m^2^ flashes, whereas photopic ERGs were obtained with 7.6 cd-s/m^2^ flashes under light-adapted conditions using a rod-saturating white background light (scotopic scale bar: x-axis = 20 ms, y-axis = 200 μV; photopic scale bar: x-axis = 20 ms, y-axis = 20 μV). *B*, the intensity-response plot of the scotopic “a”-wave response from ret-Msi1-/- mice. *C*, the plot of the rod photoreceptor “a”-wave response at light intensity of 1cd∗s/m^2^ from ret-Msi1-/- mice against the age of the mouse during which the ERG was recorded. *D*, the intensity-response curve of the scotopic “a”-wave response from ret-Msi2-/- mice. *E*, the plot of the rod photoreceptor “a”-wave response at light intensity of 1cd∗s/m^2^ from ret-Msi2-/- mice plotted against the age of the mouse during which the ERG was recorded. For panels *B–E*, the significance levels of pairwise *t*-test after FDR correction are indicated as follows: ∗*p*-value < 0.05; ∗∗*p*-value < 0.01; ∗∗∗*p*-value < 0.001. FDR, false discovery rate; P, postnatal day.

To determine if the Musashi proteins are required for photoreceptor function, we performed electroretinographic (ERG) recordings of the *Musashi* conditional KO mice at P16 and monitored for changes in retinal function up to P180. Fig. 2*A* shows the representative scotopic and photopic ERG waveforms of the *ret-Msi1-/-*, *ret-Msi2-/-*, and *ret-Msi1-/-:Msi2-/-* mice at P16 immediately after mice open their eyes (23). When both *Musashi* genes are removed, no scotopic or photopic response remains as shown by the absence of conspicuous “a” waves and “b” waves (Fig. 2*A*). However, significant photoreceptor function remains in the *ret-Msi1-/-* and *ret-Msi2-/-* single-KO mice. We characterized the photoreceptor function of the *ret-Msi1-/-* and *ret-Msi2-/-* mice further to see if there was a progressive loss of vision as the mice aged (Fig. 2, *B*–*E*). In *ret-Msi1-/-* mice, there was a statistically significant reduction in photoreceptor “a”-wave amplitudes at light intensities above 0.06 cd∗s/m^2^ (Fig. 2*B*). Two-way ANOVA examining the effect of the genotype and light intensity on the "a"wave intensity showed a significant main effect of the genotype (F(1,104)= 35.88, *p*-value<0.001) and a significant interaction between the two factors (F(1,104)= 25.78, *p*-value<0.001). This reduction in the photoreceptor “a”-wave amplitude was unchanged over time (repeated measures ANOVA was not significant) and persisted in *ret-Msi1-/-* mice up to P180 (Fig. 2*C*). On the other hand, *ret-Msi2-/-* mice at P16 had normal photoreceptor function at all the light intensities we tested (Fig. 2*D*). The “a”-wave amplitude began to decrease progressively in *ret-Msi2-/-* mice as they aged (Fig. 2*E*), and this became significant at P120 (repeated measures ANOVA F(6,12) = 8.15, *p*-value=0.001, η^2^_g_ = 0.74).

**Figure 3:**
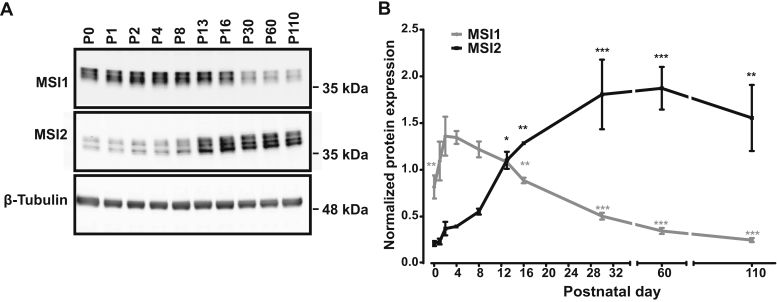
**Developmental switch in expression of MSI1 and MSI2**. *A*, representative immunoblot showing the expression of MSI1 and MSI2 in retinal tissues at indicated ages (P0-P110). Equal amounts of total protein (20 μg) were loaded in each lane. β-tubulin serves as the loading control. *B*, quantification of immunoblots shown in panel A (n = 3). All data are shown as the mean ± the SEM. One-way ANOVA with Welch’s correction F(9, 8.02) = 90.78, *p* < 0.001 for Msi1 and F(9, 7.97) = 29.28, *p* < 0.001. Significance levels relative to P4 for MSI1 and P0 for MSI2 of pairwise *t*-test after FDR correction are indicated as follows: ∗*p*-value < 0.05; ∗∗*p*-value < 0.01; ∗∗=*p*-value < 0.001. FDR, false discovery rate; P, postnatal day.

The two Musashi proteins share a high degree of sequence similarity and are proposed to be functionally redundant, yet the progression of vision loss in the single *Msi1* and *Msi2* KO mice was significantly different. We tested if changes in expression levels of the two proteins after birth may account for this discrepancy. The Western blot analysis of the Musashi protein expression levels in the retina between P0 and P110 showed a distinct pattern of expression (Fig. 3, *A* and *B*). MSI1 levels peak between P2 and P4 and remain high until P13 to P16. The time frame of high MSI1 expression includes the period of photoreceptor OS morphogenesis (Fig. 3, *A* and *B*). After eye opening, MSI1 protein expression declines (Fig. 3, *A*–*B*). MSI2 shows an inverse pattern of protein expression to that of MSI1: relatively low levels after birth that gradually increase and peak after P16 as the MSI1 protein levels decline (Fig. 3, *A* and *B*). Overall, our data show that the Musashi proteins are essential for photoreceptor function. The two proteins are partially redundant and appear to act at different time points of the retinal development.

### Intrinsic expression of Musashi in photoreceptors is crucial for photoreceptor function

**Figure 4:**
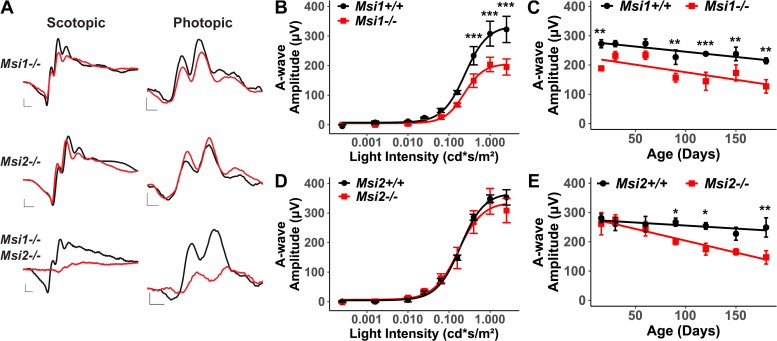
**Rod cell–specific defect of the double Msi1 and Msi2 KO mice.***A*, representative scotopic and photopic electroretinograms (ERGs) from the rod-Msi1-/-, rod-Msi2-/-, and rod-Msi1-/-: Msi2-/- mice at P16. Scotopic ERGs were obtained after overnight dark adaptation using 0.151 cd-s/m^2^ flashes, whereas photopic ERGs were obtained with 7.6 cd-s/m^2^ flashes under light-adapted conditions using a rod-saturating white background light (scotopic scale bar: x-axis = 10 ms, y-axis = 100 μV; photopic scale bar: x-axis = 10 ms, y-axis = 20 μV). *B*, intensity response plot of the scotopic “a”-wave from rod-Msi1-/- mice. *C*, the plot of the rod photoreceptor “a”-wave response at light intensity of 1cd∗s/m^2^ from rod-Msi1-/- mice against the age of the mouse during which the ERG was recorded. *D*, the intensity response plot of the scotopic “a”-wave response from rod-Msi2-/- mice. *E*, the plot of the rod photoreceptor “a”-wave response at light intensity of 1cd∗s/m^2^ from rod-Msi2-/- mice against the age of the mouse during which the ERG was recorded. For panels *B–E*, the significance levels of pairwise *t*-test after FDR correction are indicated as follows: ∗*p*-value < 0.05; ∗∗*p*-value < 0.01; ∗∗∗*p*-value < 0.001. FDR, false discovery rate; P, postnatal day.

We next sought to determine if the phenotype of the *ret-Msi-/-* mice was due to the absence of Musashi protein expression in photoreceptors or if the deletion of Musashi in other retinal cell types or retinal progenitors was contributing to the loss of vision. To this end, we generated rod-specific *Musashi* conditional KO mice by crossing *Musashi* floxed mice with mice hemizygous for the *Nrl-Cre* transgene where the *Nrl* promoter activates Cre expression in rod photoreceptors (24). Throughout this work, we refer to the *Musashi* floxed mice that are hemizygous for the *Nrl-Cre* transgene as *rod-Msi-/-* mice. We used ERG to analyze the retinal function of the KO mice after ablation of the *Musashi* genes in rods (Fig. 4, *A*–*E*). Figure 4*A* shows the scotopic and photopic ERG waveforms of the *rod-Msi1-/-*, *rod-Msi2-/*-, and *rod-Msi1-/-:Msi2-/-* mice at P16. As observed in the *ret-Msi1-/-:Msi2-/-* mice, no significant rod function was observed in the *rod-Msi1-/-:Msi2-/-* mice at P16, which is demonstrated by the absence of conspicuous “a” wave under scotopic testing conditions (Fig. 4*A*). We examined the *rod-Msi1-/-* and *rod-Msi2-/-* single-KO mice to see if the photoresponse phenotype was comparable to that obtained from the *ret-Msi1-/-* and *ret-Msi2-/-* mice. In *rod-Msi1-/-* mice at P16, there was a reduction in photoreceptor “a”-wave amplitudes at multiple light intensities (Fig. 4*B*). Two-way ANOVA examining the effect of the genotype and light intensity on the "a"-wave intensity showed a significant main effect of the genotype (F(1,50)= 7.85, *p*-value < 0.01) and a significant interaction between the two factors (F(1,50)= 6.82, *p*-value < 0.05). This reduction in “a”-wave amplitude persisted as these mice aged up to P180 (Fig. 4*C*). Contrarily, P16 *rod-Msi2-/-* mice had no changes in photoreceptor function at all the light intensities examined (Fig. 4*D*). As observed in the *ret-Msi2-/-* mice, the “a”-wave amplitude began to decrease progressively as these mice aged (Fig. 4*E*), and this decrease became statistically significant at P90 (repeated measures ANOVA F(6,6) = 6.39, *p*-value<0.05, η^2^_g_ = 0.75). The similar phenotypes of the *ret-Msi* and *rod-Msi* KO mice show that the intrinsic expression of Musashi proteins in photoreceptors is crucial for their function and that deletion of Musashi proteins in other cell types likely does not contribute significantly to the phenotype observed in the *ret-Msi-/-* mice. Therefore, throughout the rest of our studies, we focus on the *ret-Msi1-/-:Msi2-/-* mouse model for our experiments because there is a compensation in the function occurring between MSI1 and MSI2 in the single-KO mice and to avoid confounding results that might be obtained when *Msi1* and *Msi2* are deleted only in rod but not cone photoreceptors.

### Progressive neuronal degeneration in the absence of the Musashi proteins

**Figure 5:**
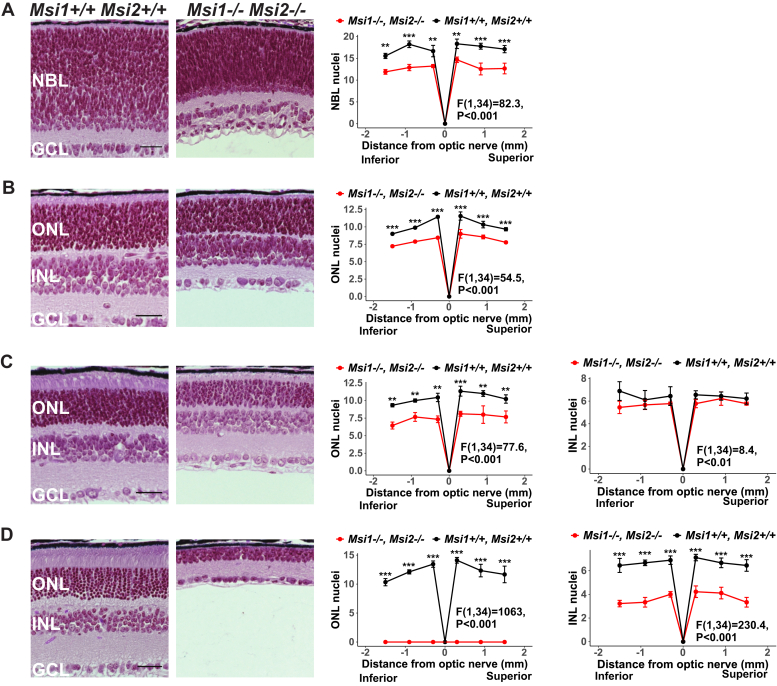
**Retinal cell death occurs in the absence of the Musashi proteins.** Left: Brightfield microscopic images of H&E-stained retinal cross sections from the ret-Msi1-/-: Msi2-/- mice at P5 (*A*), P10 (*B*), P16 (*C*), and P180 (*D*). Right: The spider plot of the indicated layer thickness at six regions from the inferior to superior retina in the ret-Msi1-/-: Msi2-/- mice at P5 (*A*), P10 (*B*), P16 (*C*), and P180 (*D*). All data are shown as the mean ± SEM. Significance levels for one-way ANOVA are shown for each plot. Significance levels of pairwise *t*-test after FDR correction are indicated as follows: ∗∗*p*-value < 0.01; ∗∗∗*p*-value < 0.001. Scale bars are 30 μm. INL, inner nuclear layer; GCL, ganglion cell layer; P, postnatal day; NBL, neuroblast layer; ONL, outer nuclear layer.

We next wanted to examine the mechanism behind the photoreceptor dysfunction seen in the *ret-Msi1-/-:Msi2-/-* mouse model. One of the common causes of a reduced ERG is photoreceptor cell death. Therefore, we performed histological analysis of the *ret-Msi1-/-:Msi2-/-* mice at P5, P10, P16, and P180 (Fig. 5, *A*–*D*). In *ret-Msi1-/-:Msi2-/-* mice at P5, even before the neural retina has completely differentiated, there is a reduction in the neuroblast-layer thickness, which was quantified across the superior-inferior axis (Fig. 5*A*, the left and right panels). There is also a more disordered arrangement of neuroblast-layer nuclei in *ret-Msi1-/-:Msi2-/-* mice, with cells more tightly packed together than their littermate control (Fig. 5*A*, left panel). At P10, the outer nuclear layer (ONL), inner nuclear layer (INL), and ganglion cell layer of the retina all form in *ret-Msi1-/-:Msi2-/-* mice, but there is a reduction in the number of layers of photoreceptor nuclei (Fig. 5*B*, the left and right panels). At P16, the number of layers of ONL nuclei continues to decrease, suggesting that photoreceptor cell death is occurring (Fig. 5*C*, the left and middle panels). At this age, ANOVA shows statistically significant difference in the number of layers of INL nuclei (Fig. 5*C*, the left and right panels) between the WT and KO animals. However, the difference is of small amplitude and the pairwise comparison at different retinal locations was not statistically significant. By the age of 6 months, the retina of *ret-Msi1-/-:Msi2-/-* mice was severely degenerated with a complete loss of ONL nuclei in addition to a significant reduction in the number of layers of INL nuclei (Fig. 5*D*, the left, middle, and right panels).

### The Musashi proteins are crucial for photoreceptor OS and axoneme development

**Figure 6:**
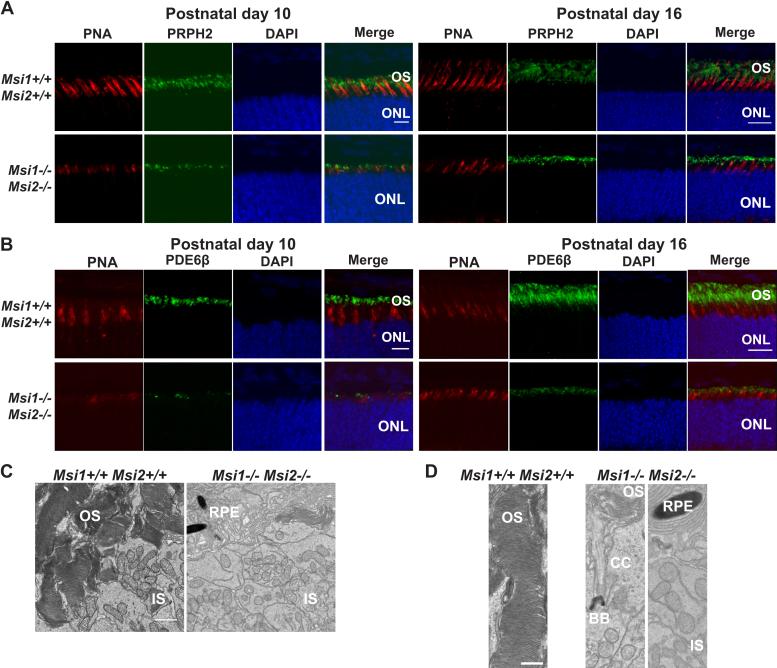
**Abnormal development of OS in the absence of MSI1 and MSI2.***A*, immunofluorescence microscopy images of retinal cross sections from the ret-Msi1-/-: Msi2-/- mice at P10 (*left*) and P16 (*right*) stained with anti–peripherin-2 antibody (PRPH2: OS marker–*green*) and peanut agglutinin (PNA: cone OS marker–*red*) along with a DAPI nuclear counterstain (*blue*). Scale bar = 20 μm. *B*, immunofluorescence microscopy images of retinal cross sections from the ret-Msi1-/-: Msi2-/- mice at P10 (*left*) and P16 (*right*) stained with anti–phosphodiesterase-6β antibody (PDE6β: rod OS marker–*green*) and peanut agglutinin (PNA: cone OS marker–*red*) along with a DAPI counterstain (*blue*). Scale bar = 20 μm. *C*, low-magnification transmission electron microscopy images of ultrathin retinal sections from ret-Msi1-/-: Msi2-/- mice at P10 visualizing the boundary between the OS and IS showing the lack of typical outer segments in the absence of the Musashi proteins. Scale bar = 2 μm. *D*, high-magnification transmission electron microscopy images of ultrathin retinal sections from ret-Msi1-/-: Msi2-/- mice at P10 visualizing the boundary between the OS and IS showing that the OS either does not form (*far right*) or is dysmorphic (*middle*) in the absence of the Musashi proteins. Scale bar = 1 μm. BB, basal body; CC, connecting cilium; DAPI, 4′,6-diamidino-2-phenylindole; IS, inner segment; ONL, outer nuclear layer; OS, outer segment; RPE, retinal pigment epithelium; P, postnatal day.

Photoreceptor cells are present in the *ret-Msi1-/-:Msi2-/-* mice as indicated by the well-defined ONL (Fig. 1*C*). We therefore examined the structure of the OS in *ret-Msi1-/-:Msi2-/-* mice at P16 by immunofluorescence microscopy using three different OS markers, anti–peripherin-2 (PRPH2: OS marker), anti–phosphodiesterase-6β (PDE6β: rod OS marker), and peanut agglutinin (PNA: cone OS marker). After staining retinal cross sections from *ret-Msi1-/-:Msi2-/-* mice with PRPH2 and PNA, we observed a severe shortening of the photoreceptor OS at P10 and P16 (Fig. 6*A*). This result was not limited to PRPH2, as staining with the rod OS marker PDE6β demonstrated the same phenotype (Fig.6*B*). The OS of cone photoreceptors also appears to be severely shortened as shown by the abnormal PNA staining (Fig. 6, *A* and *B*). Finally, no mislocalization of PDE6β or PRPH2 is found in the ONL or inner segment of *ret-Msi1-/-:Msi2-/-* mice, suggesting that although the Musashi proteins are required for OS formation, they are not regulating trafficking or localization of OS-resident proteins (Fig. 6*B*).

Using transmission electron microscopy, we imaged ultrathin retinal sections from *ret-Msi1-/-:Msi2-/-* mice at P10 when the OS begins to elaborate. When examining the OS–IS boundary in *ret-Msi1-/-:Msi2-/-* mice by electron microscopy, we observed very little, if any, conspicuous OS (Fig. 6*C*). Instead, the IS of the *ret-Msi1-/-:Msi2-/-* mice appears to come in direct contact with the RPE (Fig. 6, *C* and *D*). At higher magnification, the photoreceptors of *ret-Msi1-/-:Msi2-/-* mice displayed either no OS or aberrant and undersized OS (Fig. 6*D* the left, middle, and right panels).

**Figure 7:**
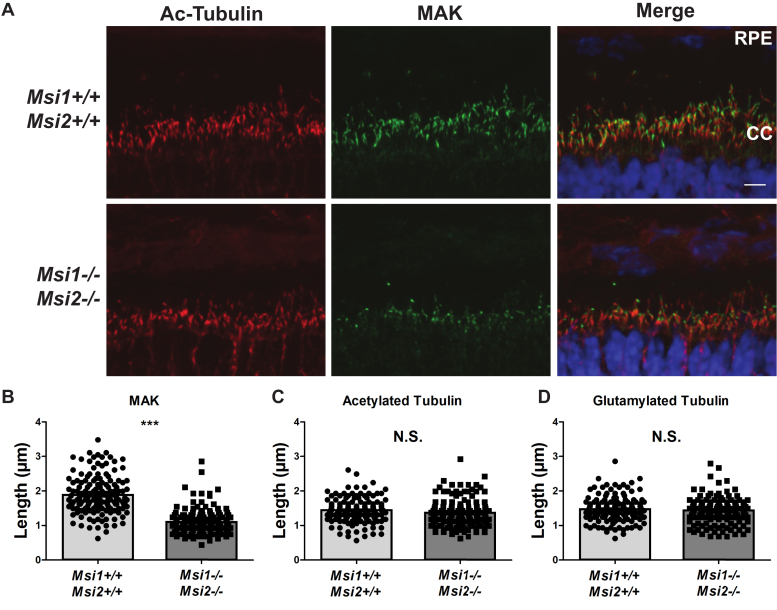
**The Musashi proteins are crucial for photoreceptor axoneme development.***A*, immunofluorescence microscopy images of retinal cross sections from the ret-Msi1-/-: Msi2-/- mice at P10 stained with acetylated α-tubulin antibody (Ac-tubulin: *red*) and male germ cell–associated kinase antibody (MAK: *green*) along with DAPI counterstain (*blue*). The scatter bar plot showing the distribution of length measurements for the photoreceptor axoneme by MAK staining (*B*) and connecting the cilium by Ac-tubulin staining (*C*) and glutamylated tubulin staining (*D*). Retinal sections were obtained from P10 Musashi KO mice and littermate controls. The scale bar is 5 μm. CC, connecting cilium; DAPI, 4′,6-diamidino-2-phenylindole; ONL, outer nuclear layer; P, postnatal day; RPE, retinal pigment epithelium. ∗∗∗*p*-value < 0.001.

To examine the structure of the CC and the axoneme, we stained retinal cross sections from *ret-Msi1-/-:Msi2-/-* mice at P10 using antibodies directed against the established markers of murine CC (glutamylated and acetylated tubulin) and axoneme (male germ cell–associated kinase [MAK]) (25, 26, 27, 28). Probing with glutamylated and acetylated α-tubulin antibodies showed that there were no changes in the length of the CC (Fig. 7, *A*, *C* and *D*). Contrarily, staining with the anti-MAK antibody showed a substantial reduction in the length of the axoneme accompanied with punctate staining, suggesting a severe structural defect of the axoneme (Fig. 7, *A* and *B*).

### The Musashi proteins promote splicing of photoreceptor-specific exons

**Figure 8:**
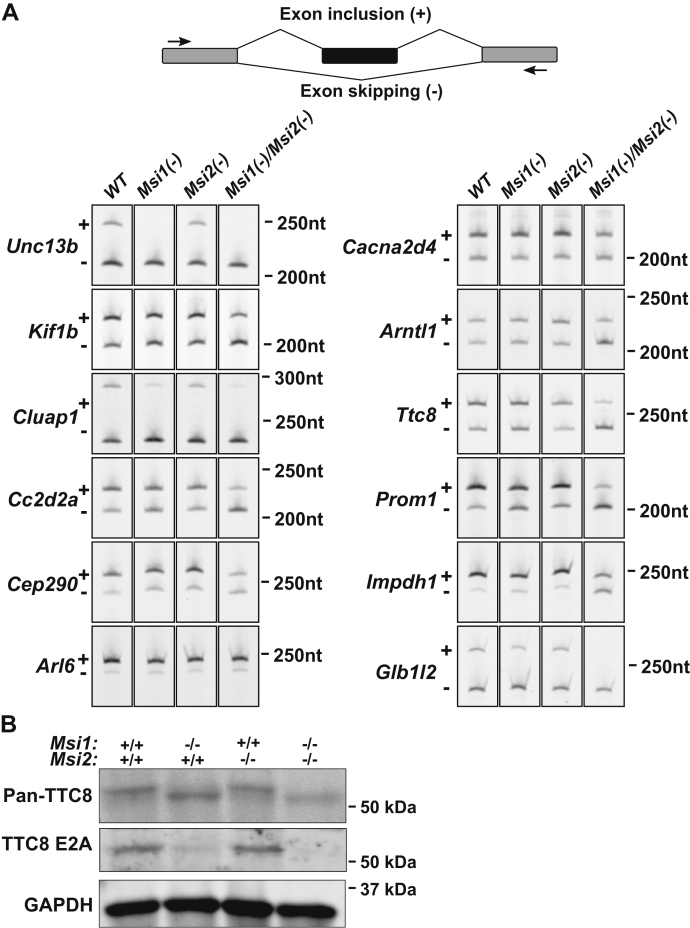
**The Musashi proteins regulate alternative splicing of their target transcripts**. *A*, RT-PCR splicing assay. The schematic of the primer design is shown on *top*. All analyzed exons are simple cassettes, and the splicing was analyzed using primers placed in the flanking constitutive exons. The RT-PCR amplification results are shown for eleven exons previously reported by us to be photoreceptor specific along with alternative exon in Arl6 which is retinal, but not photoreceptor specific. *B*, immunoblot of retinal lysates from ret-Msi1-/-, ret-Msi2-/-, and ret-Msi1-/-: Msi2-/- mice. After probing with the pan-TTC8 antibody (*top*), a change in the migration of the TTC8 protein is observed in the absence of MSI1 and MSI2, suggesting that the peptide encoded by Exon 2A was not included. When probing with the TTC8 E2A antibody (*middle*), photoreceptor-specific isoform of TTC8 was not observed in the absence of MSI1 and MSI2. TTC8, tetratricopeptide repeat domain 8.

Our previous studies suggested that the Musashi proteins are regulating alternative splicing of their target pre-mRNAs in vertebrate photoreceptors (8). To test if the Musashi proteins are responsible for the inclusion of photoreceptor-specific exon, we analyzed the splicing in *ret-Msi1-/-:Msi2-/-* mice of eleven transcripts that we previously showed to express photoreceptor-specific isoforms (Fig. 8). We also analyzed the splicing of Arl6, which contains an alternative exon that is included in all retinal cells at high levels. We witnessed a universal reduction in alternative exon inclusion in *ret-Msi1-/-:Msi2-/-* mice for all tested transcripts (Fig. 8*A*). The inclusion of different exons was reduced to varying degree depending on the transcript. Exons in the Ttc8, Glb1l2, Unc13b, Cc2d2a, Impdh1, and Prom1 showed major reduction in their inclusion levels in the *ret-Msi1-/-:Msi2-/-* mice. Single *Msi2* KO had little effect on splicing. *Msi1* KO had moderate effect on the splicing of some exons. One exception is the alternative exon in Unc13b, whose splicing was completely abolished in the *ret-Msi1-/-* mouse. We also analyzed isoform expression at the protein level for tetratricopeptide repeat domain 8 (TTC8) because we had an antibody that specifically recognizes the photoreceptor-specific isoform. TTC8 also referred as Bardet–Biedl syndrome protein is part of the BBSome complex that is known to play an important role in photoreceptor OS morphogenesis (29, 30). We used two different antibodies, a pan-antibody that recognizes all TTC8 protein isoforms and the other that recognizes the photoreceptor-specific isoform of Ttc8 by binding the epitope encoded by Exon 2A (the photoreceptor-specific exon of *Ttc8*) (Fig. 8*B*). After probing retinal lysates from the *ret-Msi1-/-:Msi2-/-* mice with the pan-TTC8 antibody, we observed faster migration of the TTC8 protein than that of the littermate control, suggesting that the Exon 2A was not included (Fig. 8*B*). Concordantly, when probing for the photoreceptor-specific isoform of TTC8 using the Ttc8 Exon 2A antibody, we saw the absence of this isoform in *ret-Msi1-/-:Msi2-/-* mice (Fig. 8*B*). Taken together, these results demonstrate that the Musashi proteins are required for the inclusion of photoreceptor-specific alternative exons.

## Discussion

### MSI1 and MSI2 are required for photoreceptor morphogenesis but not specification

Our data show the requirement for MSI1 and MSI2 in photoreceptor cells. Double KO of *Msi1* and *Msi2* in retinal progenitors results in complete loss of vision. Two lines of evidence demonstrate that this loss of vision is due to a defect in photoreceptor morphogenesis, rather than early developmental defects. First, the specification of retinal progenitors to photoreceptor cells was not affected by loss of Musashi. The retina of the KO mice had laminated nuclear layers indicating normal development of the retina. The photoreceptor cells retained their characteristic morphology and expressed cell type–specific proteins such as peripherin and PDE6. Importantly, removal of *Msi1* and *Msi2* in rod photoreceptors driven by *Nrl-Cre* caused loss of scotopic photoresponse. Thus, the vision phenotype is not due to impairment of the early stages of retinal development and is caused by a defect specific to photoreceptor cells.

Morphological examination by electron microscopy and immunofluorescence showed that the OS of the photoreceptors lacking Musashi is either missing or is stunted and disorganized. In addition, axoneme was shortened. In contrast, the CC has normal length and did not have obvious defects. Trafficking of PDE6 and peripherin through the CC also appears to be unaffected, and the two proteins localize to the stunted OS wherever one is present. Taken together, our findings demonstrate a requirement for Musashi in the morphogenesis and function of the photoreceptor OS that appears not to affect protein trafficking.

### Musashi is needed for inclusion of photoreceptor-specific exons

RT-PCR analysis of alternative splicing in the retina of *Msi1* and *Msi2* KO mice showed that inclusion of photoreceptor-specific exons in the mature transcripts is dependent on the Musashi proteins. We confirmed this finding using immunoblotting with an antibody that recognizes photoreceptor-specific isoform of TTC8. We have previously shown that the effect of Musashi on Ttc8 splicing is direct and requires binding of Musashi downstream of the alternative exons (8). Currently, we do not have sufficient data to show direct control of splicing of the other exons we show to be dependent on Musashi. The effect of *Msi1* KO on splicing is stronger than that of Msi2 knockdown. It remains to be determined if this observation reflects a dominant role for MSI1 in splicing control or derives from the timing of the embryonic KO of the two genes relative to the postnatal developmental switch from MSI1 to MSI2 expression in the retina. Our data demonstrate for the first time that Musashi regulates splicing *in vivo* and impacts dramatically the inclusion levels of the exons it controls. This is a novel role for Musashi that is distinct from it known function in controlling translation in the cytosol.

### Functional redundancy and developmental switch within the Musashi protein family

In vertebrates, the Musashi protein family consists of two paralogues, MSI1 and MSI2, which have a high degree of sequence identity and have arisen from a gene duplication event (10, 31). The RBDs of MSI1 and MSI2 have approximately 90% sequence identity and recognize the same UAG sequence motif *in vitro* and *in vivo* (32, 33, 34, 35). The high degree of similarity suggests that the two proteins are likely to be functionally redundant when coexpressed in the same cells. Indeed, we observed only minor reductions in visual function after the loss of either MSI1 or MSI2 alone, whereas the combined loss of MSI1 and MSI2 resulted in a complete loss of visual function (Fig. 2). Similarly, inclusion of photoreceptor-specific exons is promoted by both proteins, and the double KO produces a stronger effect on splicing than the KOs of either *Msi1* or *Msi2.* The functional redundancy in photoreceptor cells that we observe is in agreement with previous reports of redundancy between MSI1 and MSI2 in other cell types (12, 13).

Despite the proposed functional redundancy between the two Musashi proteins, the phenotype of the single *Msi1* and *Msi2* KO mice shows distinct progression of vision loss. *Msi1* KO mice have reduced vision at birth, followed by minor decline as the animals age. This decline is unlikely to be associated with the lack of Musashi, as it tracks the normal reduction in visual response observed in the WT controls. In contrast, *Msi2* KO mice do not show significant visual defect at the time of eye opening (P16), but their vision progressively deteriorates with age. This difference in the phenotypes may reflect an incomplete redundancy in the molecular properties of two proteins. Alternatively, the difference in phenotypes can be explained by the developmental timing of the MSI1 and MSI2 protein expression. A burst in MSI1 protein expression precedes the critical period for rod photoreceptor OS morphogenesis between birth and eye opening, and MSI1 levels remain high until the eyes open at P16. The MSI1 expression begins a gradual decline at P13, and the MSI1 protein is replaced by an increase in MSI2 levels. These data show distinct roles for MSI1 and MSI2 in photoreceptor morphogenesis and photoreceptor maintenance, respectively. The developmental switch we observe raises the question of potential functional differences in the two Musashi proteins that require MSI1 expression during photoreceptor morphogenesis and MSI2 for photoreceptor maintenance.

Our work highlights roles for MSI1 and MSI2 in photoreceptor morphogenesis and survival. An interesting aspect of the function of the Musashi proteins in the retina is their apparently mutually exclusive roles at different stages of development. At early stages of development, MSI1 and MSI2 support the renewal and proliferation of retinal precursor cells. At late stages of retinal development and in the adult retina, MSI1 and MSI2 are required for morphogenesis of the differentiated photoreceptor cells and survival of mature neurons. Our studies point to the need for MSI in controlling the alternative splicing in photoreceptor cells. It is important to note that the canonical function of the Musashi proteins is to control mRNA translation in the cytosol (36, 37), where they can either block or enhance translation of mRNA depending on the cellular context (38, 39, 40, 41, 42, 43). Future studies will be aimed at determining the mechanism(s) for the need for Musashi in vision and the regulation of the developmental switch between MSI1 and MSI2.

## Materials and methods

### Generation of mice and genotyping

Mice carrying floxed alleles for *Msi1* and *Msi2* were provided by Dr Christopher Lengner from the University of Pennsylvania. *Six3-Cre* transgene or *Nrl-Cre* transgenes were used to delete the floxed alleles in the developing retina or rod photoreceptors (Stock Nos. 019755, 028941, Jax labs). All mouse lines in this study are in C57 Black 6/J background (https://www.jax.org/strain/000664) and were devoid of naturally occurring *rd1* and *rd8* alleles (44, 45). Male mice hemizygous for the *Six3-Cre* transgene or *Nrl-Cre* transgene and floxed for *Msi1*, *Msi2*, or both *Msi1* and *Msi2* were mated with female mice floxed for *Msi1*, *Msi2*, or both *Msi1* and *Msi2* to obtain experimental KO mice and littermate control. The offspring of breeding pairs were genotyped using PCR from ear biopsies. The *Msi1* WT and floxed alleles were identified using following primers: 5′-CGG ACT GGG AGA GGT TTC TT-3′ and 5′-AGC TCC CCT GAT TCC TGG T-3′. The *Msi2* WT and floxed alleles were identified by using following primers: 5′-GCT CGG CTG ACA AAG AAA GT-3′ and 5′-TCT CCT TGT TGC GCT CAG TA-3′. The presence of the *Six3-Cre, Nrl-Cre* transgene, and *Cre recombinase* was determined using following primers respectively: 5′-CCC AAA TGT TGC TGG ATA GT-3′ and 5′-CCC TCT CCT CTC CCT CCT-3′, 5′-TTT CAC TGG CTT CTG AGT CC-3′ and 5′-CTT CAG GTT CTG CGG GAA AC-3′, and 5′-CCT GGA AAA TGC TTC TGT CCG-3′ and 5′-CAG GGT GTT ATA AGC AAT CCC-3′.

All experiments were conducted with the approval of the Institutional Animal Care and Use Committee at West Virginia University. All experiments were carried out with adherence to the principles set forth in the Association for Research in Vision and Ophthalmology Statement for the Ethical Use of Animals in Ophthalmic and Vision Research that advocates the use of the minimum number of animals per study needed to obtain statistical significance.

### Electroretinography, immunoblotting, and RT-PCR

Electroretinography, immunoblotting, and RT-PCR were conducted using previously described protocol from our laboratory (8, 46, 47). Primers used for splicing analysis of photoreceptor-specific exons were reported previously (8). *Msi1* and *Msi2* RNA levels across mouse tissues were quantified by RT-PCR and normalized to the geometric average of two reference genes, *B2m* and *Gusb*. The primers used in the quantitative RT-PCR analysis are as follows: m-qMSi1-F AGTTCGGGGAGGTGAAAGAG, m-qMsi1-R CTGTGCTCTTCGAGGAAAGG, m-qMsi2-F CCCAAAAGTTGCATTTCCTC, m-qMsi2-R CATCAGCATCGCATCCTCTA, mB2M_qF CTCGGTGACCCTGGTCTTTC, mB2M_qR GGATTTCAATGTGAGGCGGG, Gusb_qF ACGTATTCTTTACGTTTCTG, and Gusb_qR TTGAGAACTGGTATAAGACG.

### Immunofluorescence microscopy

Immunofluorescence microscopy was carried out using a modified procedure in our laboratory (46, 47). Briefly, eyes were enucleated, and the cornea and lens were discarded. After dissection, eyes were fixed by immersion in 4% paraformaldehyde in PBS for 1 h. After washing the eyes in PBS three times for 10 min each, they were dehydrated by overnight incubation in 30% sucrose in PBS. Eyes were then incubated in a 1:1 solution of optimal cutting temperature compound:30% sucrose in PBS for 1 h and frozen in optimal cutting temperature compound (VWR). The frozen tissues were sectioned using a Leica CM1850 cryostat for collecting serial retinal sections of 16-μm thickness. The retinal cross sections were then mounted onto Superfrost Plus microscope slides (Fisher Scientific). Slide sections were then washed and permeabilized with PBS supplemented with 0.1% Triton X-100 (PBST) and incubated for 1 h in a blocking buffer containing 10% goat serum, 0.3% Triton X-100, and 0.02% sodium azide in PBS. Retinal sections were then incubated with a primary antibody in a dilution buffer containing 5% goat serum, 0.3% Triton X-100, 0.02% sodium azide, and primary antibody at 1:500 dilution in PBS overnight at 4 ^o^C followed by three 5-min washes using PBST. Sections were then incubated in the same dilution buffer containing secondary antibodies and 4′,6-diamidino-2-phenylindole at 1:1000 for 1 h. Slides were washed with PBST three times for 5 min each before treating with the ProLong Gold Antifade reagent (Thermo Fisher) and securing the coverslip. The images were collected using a Nikon C2 confocal microscope.

### Retinal histology of the mouse models

After euthanasia, eyes were enucleated using C-shaped forceps after marking the superior pole and incubated in Z-fixative for >48 h before shipment and tissue processing by Excalibur Pathology Inc (Norman, OK). The embedding, serial sectioning, mounting, and H&E staining were performed by Excalibur Pathology. A Nikon C2 microscope equipped with Elements software was used to image the slides.

### Transmission electron microscopy

After euthanasia, a C-shaped forceps was used to enucleate the eye, and the cornea was discarded (46, 47). The eyes were then incubated in a fixative solution containing 2.5% glutaraldehyde and 2% paraformaldehyde in 100-mM sodium cacodylate buffer at pH 7.5 for 45 min before removal of the lens. After lensectomy, eyes were placed back into fixative for 72 h before shipment, tissue processing, and imaging at the Robert P. Apkarian Integrated Electron Microscopy Core at Emory University.

### Antibodies and stains

The following primary antibodies were used throughout our studies: rat anti-MSI1 (1:1000; MBL International Cat# D270-3, RRID:AB_1953023), rabbit anti-MSI2 (1:2000; Abcam Cat# ab76148, RRID:AB_1523981), mouse anti–α-tubulin (1:10,000; Sigma-Aldrich Cat# T8328, RRID:AB_1844090), rhodamine peanut agglutinin (1:1000; Vector Laboratories Cat# RL-1072, RRID:AB_2336642), rabbit anti–peripherin-2 (1:2000), which was a kind gift by Dr Andrew Goldberg from Oakland University, rabbit anti-PDE6β (1:2000; Thermo Fisher Scientific Cat# PA1-722, RRID:AB_2161443), mouse anti-acetylated α-tubulin (1:1000; Santa Cruz Biotechnology Cat# sc-23950, RRID:AB_628409), guinea pig anti-MAK (1:500; Wako, Cat# 012-26441, RRID:AB_2827389), mouse anti-glutamylated tubulin (1:500; AdipoGen Cat# AG-20B-0020B, RRID:AB_2490211), mouse anti-Ttc8 (1:1000; Santa Cruz Biotechnology Cat# sc-271009, RRID:AB_10609492), rabbit anti-Ttc8 Exon 2A (1:1000; Peter Stoilov, West Virginia University, Cat# Anti-Bbs8 exon 2A, RRID:AB_2827390), mouse anti-GAPDH (1:10,000; Fitzgerald Industries International Cat# 10R-G109a, RRID:AB_1285808), and 4′,6-diamidino-2-phenylindole (nuclear counterstain; 1:1000; ThermoFisher, Waltham, MA).

### Statistical analysis

Unless otherwise stated, the data are presented as the mean of at least three biological replicates with error bars representing the SEM. Statistical significance two-way comparisons was determined by homoscedastic, two-tailed unpaired *T*-test. Statistical significance of multiple comparisons was determined by ANOVA as indicated in the Results section. Pairwise *t*-test with pooled SD was used for post hoc two-way comparisons. *p*-values from the pairwise *t*-tests are reported after false discovery rate correction for multiple comparisons.

## Data availability

All data presented are contained within the manuscript.

## Conflict of interest

The authors declare that they have no conflicts of interest with the contents of this article.
